# LcrQ Blocks the Role of LcrF in Regulating the Ysc-Yop Type III Secretion Genes in *Yersinia pseudotuberculosis*

**DOI:** 10.1371/journal.pone.0092243

**Published:** 2014-03-21

**Authors:** Lamei Li, Huan Yan, Lipeng Feng, Yunlong Li, Pei Lu, Yangbo Hu, Shiyun Chen

**Affiliations:** Center for Emerging Infectious Diseases, Wuhan Institute of Virology, Chinese Academy of Sciences, Wuhan, China; Indian Institute of Science, India

## Abstract

Pathogenic *Yersinia* species employ the Ysc-Yop type III secretion system (T3SS) encoded by a highly conserved pYV virulence plasmid to export the virulence effectors into host cells. The Ysc-Yop T3SS is tightly regulated by multiple contributing proteins that function at different levels. However, systematic transcriptional regulation analysis of Ysc-Yop T3SS is lacking and the detailed mechanism under this regulation process is still elusive. Aimed at systematically characterizing transcriptional regulations of all T3SS genes in *Y. pseudotuberculosis*, we amplified 97 non-coding fragments from the pYV plasmid and analyzed transcriptional responses of the T3SS genes under different growth conditions. Transcriptions of T3SS genes were induced at 37°C and genes encoding T3SS effectors were highly induced by further depletion of Ca^2+^. The temperature induced gene transcription process is mediated by modules encoded on the chromosome, while the Ca^2+^ depletion-induced process is controlled by the positive regulatory protein LcrF as well as the negative regulatory protein LcrQ. In this process, LcrQ shares the same targets with LcrF and the effect of LcrQ is dependent on the presence of LcrF. Furthermore, over-expression of LcrF showed the same phenotype as that of the *lcrQ* mutant strain and intracellular amount balance of LcrQ and LcrF is important in T3SS regulation. When the expression level of LcrF exceeds LcrQ, expression of the Ysc-Yop T3SS genes is activated and *vice versa*. Together, these data support a model in which LcrQ blocks the activation role of LcrF in regulating the transcription of T3SS genes in *Yersinia*.

## Introduction

The genus *Yersinia* includes three human pathogenic bacterial species, *Y. pestis*, *Y. enterocolitica* and *Y. pseudotuberculosis*. These pathogens harbor a conserved virulence plasmid called pYV (or pCD) which encodes the Ysc-Yop type III secretion system (T3SS) [1]. When contacting with host cells, pathogenic *Yersinia* can efficiently inject effectors termed Yops (*Yersinia* outer proteins) into eukaryotic host cells with this secretion system. These translocated Yops enable bacteria to evade host immune responses by modulating host cell signaling pathways and facilitate the following infection [1]–[3].

The Ysc-Yop T3SS in *Yersinia* is both positively and negatively regulated and is induced by low Ca^2+^ conditions at 37°C *in vitro* or by direct contact with host cells *in vivo* [4], [5]. Several regulators are involved in the negative regulatory process. The LcrQ protein (or the YscM1 and YscM2 homologues in *Y. enterocolitica*) is an anti-activator which causes the feedback inhibition for Ysc-Yop T3SS gene expression. This protein is secreted outside of cells by the secretory machinery under low Ca^2+^ conditions at 37°C with the help from a chaperone protein named SycH [6]–[8]. This process relieves the repressive effect of LcrQ and results in the activation of the Ysc-Yop T3SS [7]. We have also recently characterized the role of an export apparatus component named YscV in LcrQ secretion and subsequent Ysc-Yops T3SS regulation [9]. However, the targets and roles of LcrQ during this regulatory process have not yet been fully illustrated. In addition to LcrQ, other negative regulatory elements, including SycN, YscB, YopN, TyeA and the *lcrGVH-yopBD* operon were also demonstrated to play important roles in regulating T3SS gene expression [10]–[14].

The AraC family activator LcrF (known as VirF in *Y. enterocolitica*) is the only protein from the pYV plasmid that positively regulates the transcription of T3SS genes [15]. LcrF directly binds to the promoter regions of several *yop* genes and also binds to RNA polymerase as supposed by the AraC family proteins [16]. With such interactions, LcrF enhances the binding of RNA polymerase to specific promoters to facilitate the transcription initiation. Transcription of a number of genes from the pYV plasmid, e.g., *ylpA, yadA*, *yopE*, *yopH*, *virC* and *lcrGVHyopBD* operons, are dependent on LcrF [17]–[20]. The direct bindings of LcrF to *yopE*, *yopH, virC* and *lcrGVHyopBD* have been confirmed and the ∼40 bp AT-rich region in each of these promoters is characterized to be recognized by LcrF [20].

The Ysc-Yop T3SS in *Yersinia* is regulated at both transcriptional and post-transcriptional levels. LcrF is a classical activator that controls T3SS gene expression at the transcriptional level [20]. YopD is another regulator which binds to the mRNA of several *yop* genes to accelerate mRNA degradation or inhibit the ribosome binding in gene translation [21], [22], which acts at the post-transcriptional level. The expression of *lcrF* has also been shown to be regulated at the post-transcriptional level. The mRNA structure around the ribosomal binding region of *lcrF* transcript is opened when the temperature is shifted from 26°C to 37°C [23], which would facilitate the gene translation. Several other regulators, such as the YopN-TyeA complex, also act at the post-translational level to inhibit T3SS secretion process [13], [24]. Although LcrQ negatively regulates the expression of T3SS genes, it lacks obvious DNA or RNA binding domains, and does not act by obstructing the secretion channel [25], thus the regulatory mechanism of LcrQ is not fully understood.

In this study, we systematically analyzed the responses of each gene on the pYV plasmid at the transcriptional level under different growth conditions in *Y. pseudotuberculosis* YPIII strain. By comparing the activities of each promoter, the transcriptional responses of the Ysc-Yop T3SS genes were characterized. The role of LcrQ in regulating the Ysc-Yop T3SS genes at the transcriptional level was subsequently analyzed, and its interaction with LcrF in this transcriptional regulatory process was further investigated and discussed.

## Materials and Methods

### Bacterial strains, plasmids, growth media and oligos

Bacterial strains and plasmids used in this study are summarized in **Table 1**. *Yersinia pseudotuberculosis* YPIII strains were grown in YLB medium (1% tryptone, 0.5% yeast extract, 0.5% NaCl) and *E. coli* strains were grown in Luria-Bertani (LB) medium. When appropriate, antibiotics were used at the following concentrations: nalidixic acid (Nal) 15 μg ml^−1^, chloramphenicol 30 μg ml^−1^, ampicillin 100 μg ml^−1^, kanamycin 100 μg ml^−1^. All oligos used in this study are listed in **Table S1**.

**Table 1:** The strains and plasmids used in this work.

Strain or plasmid Description* Source			
Bacteria			
***Y. pesudotuberculosis***			
YPIII		Parental strain *Y. pseudotuberculosis* YPIII, Nal^r^	Lab collection
ΔpYV		YPIII without virulence plasmid, Nal^r^	This study
Δ*lcrF*		YPIII with the deletion of *lcrF* gene, Nal^r^	This study
Δ*lcrQ*		YPIII with the deletion of *lcrQ* gene, Nal^r^	[9]
Δ*yscV*_618–644_		YPIII with deletion of a 81 bp fragment encoding amino acid 618–644 in *yscV* gene, Nal^r^	[9]
***E. coli***			
DH5α		Used for Cloning construction	Lab collection
BL21(DE3)		Used for protein expression	Novagen
S17-1 (λ-pir)		λ-*pir* lysogen of S17-1, *thi pro hsdR hsdM* ^+^ *recA* RP4 2-Tc::Mu-Kan::Tn7	Lab collection
BTH101		F^−^,*cya-99, ara*D139, *gal*E15, *gal*K16, *rps*L1^+^, *hsd*R2, *mcr*A1, *mcr*B1	Lab collection
**Plasmid**			
pDM4		Suicide vector, *mobRK2*, *oriR6K*, *sacBR*, Cm^r^	[27]
pDM4-*lcrF*m		Suicide plasmid for Δ*lcrF* construction, Cm^r^	This study
pOVR200		Plasmid for over-expressing proteins, *lac* promoter, Amp^r^	This study
pOVR-LcrF		pOVR200 carrying *lcrF* gene, Amp^r^	This study
pOVR-LcrQ		pOVR200 carrying *lcrQ* gene, Amp^r^	This study
pOVR-GST-LcrQ		pOVR200 carrying *GST*-*lcrQ* fusion, Amp^r^	This study
pKT100		Cloning vector, p15A replicon, Kan^r^	[26]
pKT-LcrF		pKT100 carrying *lcrF* gene, Kan^r^	This study
pKT-LcrQ		pKT100 carrying *lcrQ* gene, Kan^r^	This study
pKT-GST-LcrQ		pKT100 carrying *GST*-*lcrQ* fusion, Kan^r^	This study
pZT100		Promoter cloning vector, transcriptional *lacZ* fusion, Kan^r^	[9]
pKT25		Two-hybrid vector, for T25 fragment fusion, Kan^r^	[30]
pKT25-LcrF		pKT25 carrying *lcrF* gene, Kan^r^	This study
pKT25-SycH		pKT25 carrying *sycH* gene, Kan^r^	This study
pUT18		Two-hybrid vector, T18 fragment fusion, Amp^r^	[30]
pUT18-LcrF		pUT18 carrying *lcrF* gene, Amp^r^	This study
pUT18-LcrQ		pUT18 carrying *lcrQ* gene, Amp^r^	This study
pET28a		Protein expression vector, Kan^r^	Novagen
pET28a-LcrF		pET28a carrying *lcrF* gene from YPIII, Kan^r^	This study

* Nal^r^, Cm^r^, Kan^r^ and Amp^r^ represent resistance to naladixic acid, chloramphenicol, kanamycin and ampicillin respectively.

### Promoter cloning and β-galactosidase assay

The sequence of the pYV virulence plasmid in *Y. pseudotuberculosis* YPIII strain is not available. We collected and analyzed available sequences of the virulence plasmids of different *Y. pseudotuberculosis* strains from the NCBI database, e.g., pYV from IP 32953 (NC_006153.2) and IP 31758 (NC_009704.1), pYPTS01 from PB1+ strain (NC_010635.1). There are 99 annotated genes on the pYV plasmid from IP32953, 66 genes from IP31758, and 87 genes on pYPTS01 from PB1+ strain. To cover all possible genes, we chose the sequence of the pYV plasmid from IP32953 as the template to design primers (**Table S1**) for cloning of the non-coding fragments from the pYV plasmid in YPIII. All these amplified non-coding fragments were cloned into the promoter cloning vector pZT100 [9]. These clones were respectively transformed into YPIII or its derivative strains and the β-galactosidase assay was performed at least in triplicate as previously described [26] at 26°C, or 37°C or 37°C with Ca^2+^ depletion.

### RNA isolation and real-time PCR

Total RNA of *Yersinia* strains cultured at 26°C, or 37°C or 37°C with Ca^2+^ depletion was extracted with TRIzol reagent (Invitrogen) according to the manufacturer’s instruction. After treatment with RNase-free DNase I (Promerga), cDNA was obtained by reverse transcription (Random 9 mers, TaKaRa) using 2 μg of each RNA sample. Real-time PCR was performed in reactions containing 1×iTaq™ Universal SYBR Green Supermix Reagent (Bio-Rad), cDNA templates and the gene specific primers using the CFX Connect Bio-Rad system. All these assays were tested in duplicates. The mRNA level in each sample was normalized to the level of 16S rRNA gene transcript and amplification specificity was assessed using melting curve analysis. The degrees of expression change were calculated using the 2−ΔΔCt method.

### Gel-retardation assay

The gel-retardation was performed as described previously [26]. The LcrF protein was expressed in *E. coli* BL21(DE3) by pET28a plasmid carrying the *lcrF* gene from YPIII (named pET28a-LcrF), and purified by Ni-NTA resin. About 400 ng of each DNA fragment, which was amplified from the YPIII genomic DNA, was incubated with various amounts of purified LcrF protein in 20 μl of binding buffer (20 mM Tris-HCl pH 7.4, 4 mM MgCl_2_, 100 mM NaCl, 1 mM dithiothreitol, 10% glycerol, and 100 ng bovine serum albumin). A ∼200 bp fragment amplified from the non-coding region upstream of the pYV0053 gene was used as a negative control. Probe binding were performed at 37°C for 1 h, and were then loaded onto a 6.5% native polyacrylamide gel. Electrophoresis was performed in 0.5×TBE buffer on ice. The gel was stained with ethidium bromide for 20 min and scanned using a Syngene GeneGenius gel documentation system.

### Mutant construction and complementation

For Δ*lcrF* construction, two DNA fragments flanking the *lcrF* gene were amplified by PCR from the genomic DNA of YPIII using two pairs of primers: named LcrF-in-upF/R, and LcrF-in-downF/R (**Table S1**). These two fragments were mixed and then used as the template DNA in a second round of PCR with primers LcrF-in-upF and LcrF-in-downR. The PCR product was digested with *Sal*I and *Bgl*II and inserted into the suicide plasmid pDM4 [27]. This recombinant plasmid was transformed into *E. coli* S17-1 and transconjugation was performed as described [27] to obtain the Δ*lcrF* strain. To complement Δ*lcrF*, a plasmid named pOVR200 was constructed, which contains the pMB1 replicon, the *bla* gene and the *lacI* gene from pET21a (Novagen) and a *lac* promoter upstream of the multiple cloning site. The *lcrF* gene was PCR amplified from YPIII and was cloned into pOVR200 to obtain pOVR-LcrF, which was transformed into Δ*lcrF*. The pOVR200 plasmid was also transformed into Δ*lcrF* as a control.

### Over-expression and co-expression of LcrF and LcrQ

The pOVR-LcrF plasmid obtained above was transformed into YPIII to over-express LcrF protein. To over-express LcrQ protein, *lcrQ* gene was amplified from YPIII and cloned into pOVR200 to obtain pOVR-LcrQ, which was transformed into YPIII. For co-expression of LcrF and LcrQ (or GST-LcrQ), the low copy plasmid named pKT100 [26], which contains the pOVR200 compatible P15A replicon and a promoter region of the chloramphenicol resistance gene, was used to express the target proteins together with the pOVR200 plasmid.

### Yops analysis

Overnight cultures of *Yersinia* strains grown in Ca^2+^ rich medium (YLB with 2.5 mM Ca^2+^) were diluted (1:20) into fresh Ca^2+^-depleted medium (YLB containing 5 mM EGTA and 20 mM MgCl_2_) [28] and incubated at 26°C for 1 h, then shifted to 37°C to induce Yops expression and secretion. When required, isopropyl β-D-thiogalactoside (IPTG) at a final concentration of 1 mM was added into the cultures before temperature changes. After 4 h of incubation at 37°C, 20 ml of each culture was harvested and centrifuged. The wet weight of the bacterial cell pellet was measured and the supernatant was collected and filtered by 0.22 μm filter (Millipore). Proteins from the supernatant were precipitated overnight at 4°C with 10% trichloroacetic acid as described [9]. The proteins from both supernatants and pellets were dissolved in the SDS loading buffer according to the wet weight of the bacteria and resolved by SDS-PAGE. Western-blot was carried out to detect the proteins following SDS-PAGE. The horseradish peroxidase-labeled anti-rabbit antibodies and a chemiluminescence detection kit (Beyotime Institute of Biotechnology) were used to develop the reaction following the protocol provided by the manufacturer.

### pYV plasmid curing

The pYV plasmid in YPIII was cured as described by others [29]. Briefly, YPIII strain was grown on YLB plate supplemented with 20 mM of sodium oxalate, 20 mM MgCl_2_, and 0.05% Congo Red Dye at 37°C. White colonies were picked up and cultured overnight in YLB medium and then spread on Congo Red plate. After overnight culture at 37°C, a single white colony (loss of the functional pYV plasmid results in white colonies) was chosen for PCR to further confirm that the pYV plasmid was cured using five pairs of primers, which paired to different regions on the pYV plasmid.

### Bacterial two-hybrid assay

The adenylate cyclase based bacterial two-hybrid system [30] was applied in analyzing the interaction between LcrF and LcrQ proteins. Briefly, pKT25 plasmid carrying T25 fragment was used to clone *lcrF* and *sycH* genes to obtain T25-LcrF and T25-SycH. The pUT18 carrying T18 fragment was applied to clone *lcrF* and *lcrQ* genes to obtain T18-LcrF and T18-LcrQ. These two pairs of plasmids were co-transformed into *E. coli* BTH101 and were cultured in LB medium with 1 mM IPTG at 30°C for 2 days. The β-galactosidase activities were determined as described [26].

### Statistical analysis

All data for the β-galactosidase activity assays were shown as mean ± standard deviation (SD) of the results of multiple independent experiments. Student’s T test was used to compare the data between two relevant groups.

## Results

### Transcriptional responses of T3SS genes under inducible conditions

**Figure 1:**
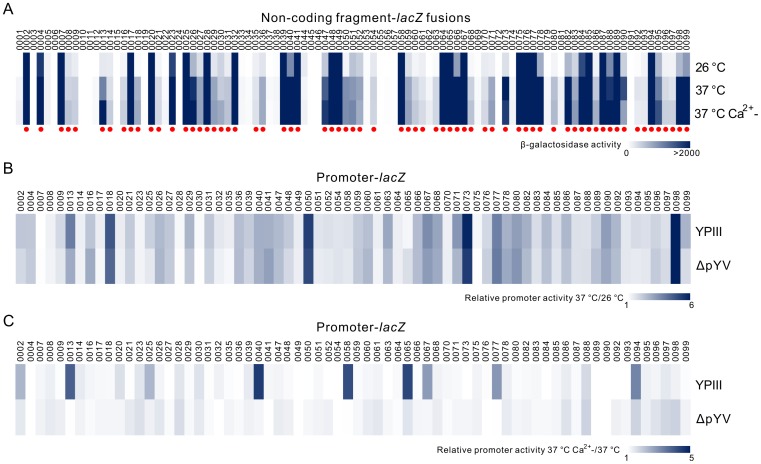
Promoter activity analyses of genes on the pYV plasmid in YPIII. (A) The promoter activities of 99 non-coding fragments at 26°C, 37°C or 37°C with Ca^2+^ depletion. Fragments with relative promoter activities higher than the pZT100 plasmid (202±12, p<0.01) at one of these conditions were marked with red dot. (B) & (C) Relative promoter activities of selected fragments in YPIII and ΔpYV strains at 26°C and 37°C (B) or at 37°C with Ca^2+^ depletion versus at 37°C (C).

To systematically characterize the transcriptional responses of Ysc-Yop T3SS genes in YPIII under T3SS inducible conditions, we compared the promoter activities of all genes from the pYV plasmid. Using primers designed from *Y. pseudotuberculosis* IP 32953 strain, we successfully amplified 96 non-coding regions from the pYV plasmid in YPIII. Only three regions upstream of pYV0042, pYV0043 and pYV0044 could not be obtained. We next sequenced the region from pYV0041 to pYV0045, a deletion at the C-terminal of the coding region of pYV0041 and a full deletion of the pYV0042 and pYV0043 genes were found in YPIII. Using this new sequence, we designed another pair of primers to amplify the non-coding region of pYV0044. In total, 97 non-coding fragments were amplified and cloned into a *lacZ* reporter vector to obtain non-coding fragment-*lacZ* fusions. The promoter activities of these non-coding fragments under T3SS non-inducible (26°C and 37°C) and inducible (37°C with Ca^2+^ depletion) conditions were subsequently analyzed. As shown in **Fig. 1A**, 68 out of the 97 non-coding fragments showed promoter activities compared with the pZT100 plasmid (202±12, p<0.01) at least under one condition. We also compared the promoter activities of these 68 non-coding fragments at 26°C and 37°C, which is one of the key factors that induces T3SS *in vitro*. As shown in **Fig. 1B**, 34 out of 68 promoters showed higher (>2 folds) activities at 37°C, suggesting some factors activate the expression of T3SS genes when bacterial cells were shifted from 26°C to 37°C.

To test if these factors are encoded by genes on the pYV plasmid, we next compared this activation in YPIII parent strain with the pYV plasmid cured strain (ΔpYV). Our results showed that induced folds at 37°C for all these promoters are similar between YPIII parent and ΔpYV strains (**Fig. 1B**), which suggests that the activation at 37°C is largely mediated by factors encoded on the chromosome. To fully characterize the transcriptional response during the T3SS inducing process, we further compared the promoter activities of these 68 fragments at 37°C and at 37°C with Ca^2+^ depletion. As shown in **Fig. 1C**, the activities of 9 promoters were induced more than 2-fold when Ca^2+^ was depleted. On the contrary to temperature changes, this activation is dependent on factors encoded on the pYV plasmid, since all these activations were strongly decreased in the ΔpYV strain (**Fig. 1C**). To further confirm the *lacZ* fusions results, we applied qRT-PCR to test the mRNA levels of *lcrG* and *lcrQ* genes in YPIII under different conditions. Being consistent with the β-galactosidase activity assay, the mRNA level of *lcrG* gene was highly induced at 37°C with Ca^2+^ depletion, and *lcrQ* gene is induced at 37°C (**Fig. S1**).

Comparing **Fig. 1B** and **Fig. 1C**, we conclude that most of the promoters activated between 37°C and the Ca^2+^ depleted conditions do not overlap, suggesting the Ysc-Yop T3SS activation process induced by 37°C and low Ca^2+^ is different. As summarized in **Table S2,** the genes induced at 37°C could be divided into four categories according to their functions: I) pYV plasmid replication; II) secretion machinery components; III) effector proteins; and IV) hypothetical proteins. On the contrary, the genes induced by Ca^2+^-depletion mainly encode effectors for Ysc-Yop T3SS (**Table S3**). Based on these analyses, we conclude that the Ysc-Yop T3SS inducing process first involves genes encoded from the YPIII chromosome, which activate the transcription of most of the secretion machinery components when the culture temperature is shifted from 26°C to 37°C, and full activation requires other key factors encoded on the pYV plasmid to maximally express the effector proteins.

### Ca^2+^-depletion induced genes are regulated by LcrF

**Figure 2:**
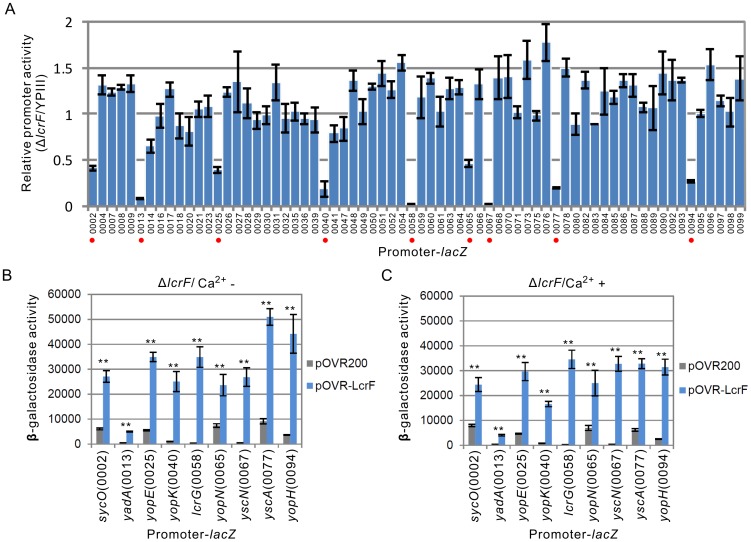
Role of LcrF in controlling the transcription of T3SS genes. (A) Relative promoter activities of genes in *lcrF* mutant (Δ*lcrF*) compared with YPIII parent strain under T3SS inducible conditions. Promoters with decreased activities were marked with red dot. (B) & (C) Effect of LcrF over-expression (pOVR-LcrF) on the promoter activities of its targets in Δ*lcrF* strain at 37°C with or without Ca^2+^. ** p<0.01.

Since LcrF protein is the only characterized transcriptional regulator from the pYV plasmid, we next aimed to explore the role of LcrF in Ca^2+^-depletion induced gene expression. To systematically analyze the regulatory targets of LcrF, We compared the promoter activities from the pYV plasmid under T3SS inducible conditions in YPIII parent and the *lcrF* mutant (Δ*lcrF*) strains. Interestingly, activities of those nine Ca^2+^-depletion induced promoters, including promoters upstream of *sycO* (pYV0002), *yadA* (pYV0013), *yopE* (pYV0025), *yopK* (pYV0040), *lcrG* (pYV0058), *yopN* (pYV0065), *yscN* (pYV0067) and *yopH* (pYV0094), were all dramatically decreased in Δ*lcrF* (**Fig 2A**), which suggests all these genes are possible targets of LcrF. To further confirm these promoters are indeed regulated by LcrF, we also compared the promoter activities in the Δ*lcrF* complementary strain. As shown in **Fig. 2B**, expression of LcrF in Δ*lcrF* successfully restored the activities of these promoters.

### Transcriptional regulation of LcrF to T3SS is not influenced by Ca^2+^

Since transcription of all the targets of LcrF is induced by Ca^2+^-depletion, we next compared the regulatory effects of LcrF under Ca^2+^ rich and depleted conditions. As shown in **Fig. 2C**, over-expression of LcrF in Δ*lcrF* induced the expression of Yops genes in the presence of Ca^2+^, which suggests the regulatory activity of LcrF is not influenced by Ca^2+^. To confirm this, we next tested the regulatory role of LcrF to its targets in the ΔpYV strain. Our results showed that expression of LcrF also increased the activities of these promoters both in Ca^2+^ rich and depleted conditions (**Fig. S2**). These data are inconsistent with the results from *Y. enterocolitica* in which the role of LcrF is not affected by Ca^2+^ [31]. However, our data demonstrated that transcription of all the promoters targeted by LcrF are strongly induced by depletion of Ca^2+^ at 37°C, which suggests other co-factor(s) may sense the change of Ca^2+^ concentration and coordinate with LcrF in regulating the Ysc-Yop T3SS.

### Role of LcrQ in the regulatory process of LcrF

**Figure 3:**
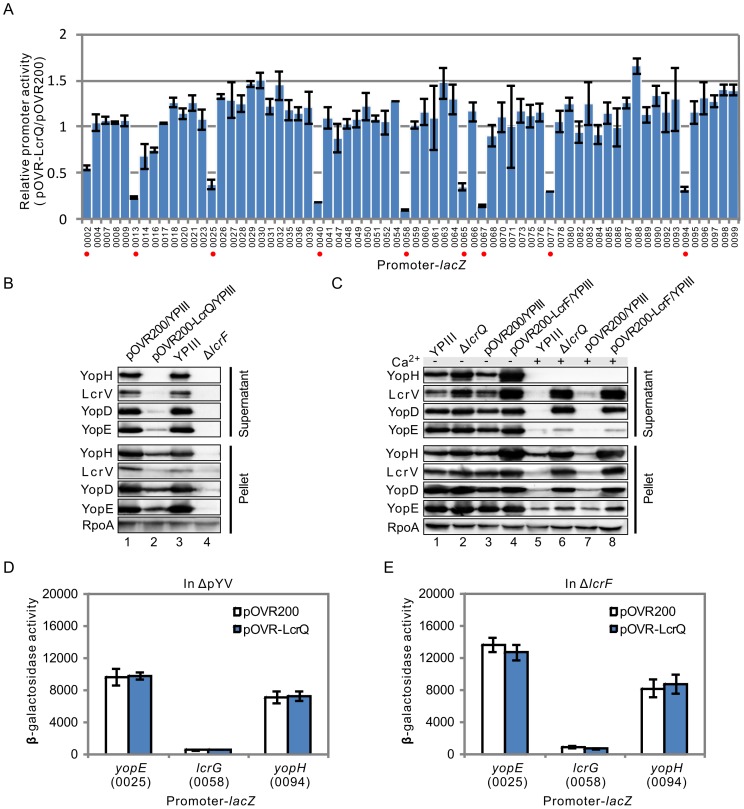
Role of LcrQ in regulating the transcription of T3SS genes. (A) Comparison of relative promoter activities of T3SS genes under T3SS inducible conditions in LcrQ over-expressed strain with YPIII carrying the pOVR200 plasmid. Promoters repressed by LcrQ were marked with red dot. (B) Secretion (Supernatant) and expression (Pellet) of T3SS effectors in LcrQ over-expressed and Δ*lcrF* strains under T3SS inducible conditions. RpoA was used as a loading control. (C) Secretion and expression of T3SS effectors in Δ*lcrQ* and LcrF over-expressed strains at 37°C with or without Ca^2+^. (D) & (E) Effects of LcrQ over-expression on the promoter activities of three LcrQ targets (*yopE*, *lcrG* and *yopH*) in YPIII ΔpYV and Δ*lcrF* strains.

The LcrQ protein globally regulates the Ysc-Yop T3SS in respond to Ca^2+^ concentration changes, but the mechanism under this regulatory role has not been illustrated. To test whether LcrQ is involved in controlling the regulatory effect of LcrF, we next screened the targets of LcrQ by comparing the promoter activities in YPIII parent and LcrQ over-expressed strains using our promoter library. Interestingly, all and only the targets of LcrF showed decreased promoter activities in the LcrQ over-expressed strain compared with those in YPIII parent strain (**Fig. 3A**), which suggests LcrQ shares the same targets with LcrF in regulating the Ysc-Yop T3SS.

To further analyze the relationship between LcrF and LcrQ in regulating the Ysc-Yop T3SS, we next compared the phenotypes of Δ*lcrF* with LcrQ over-expressed strain and LcrF over-expressed strain with Δ*lcrQ*. The expression of Yops in the LcrQ over-expressed strain was the same as that in Δ*lcrF*, which were both decreased under T3SS inducible conditions (**Fig. 3B**, lanes 2&4), and Yops secretion was also decreased as confirmed by Western blot analysis (**Fig. 3B**). Over-expression of LcrF and deletion of the *lcrQ* gene both activated Yops expression and secretion under T3SS inducible conditions (**Fig. 3C**). Moreover, deletion of the *lcrQ* gene successfully secreted LcrV and YopD proteins in the presence of Ca^2+^ at 37°C as reported by others [32], and over-expression of LcrF also promote the secretion of these two proteins (**Fig. 3C**). Together, these data suggest LcrQ and LcrF are closely related during the regulatory process.

We next compared the regulatory effects of LcrQ to its targets in YPIII parent and ΔpYV strains. As shown in **Fig. 3D**, promoter activities of the LcrQ targets were at basal levels in ΔpYV strain and over-expression of LcrQ could not repress these promoters in ΔpYV strain; however, these promoter activities were obviously inhibited by over-expressing LcrQ in YPIII parent strain (**Fig. S3**), suggesting that LcrQ represses the promoter activities activated by pYV-encoded protein. To test whether this factor is LcrF, we tested the repressive effect of LcrQ in Δ*lcrF* strain and found no repressive role to these promoters (**Fig. 3E**). It is worthy to note that the activities of these promoters in LcrQ over-expressed YPIII strain were at similar levels as that in Δ*lcrF* strain, indicating that LcrQ only represses the LcrF-activated but not the basal level promoter activities. Together, these data suggest that the repressive effect of LcrQ requires LcrF.

### Intracellular LcrQ inhibits the regulatory role of LcrF

**Figure 4:**
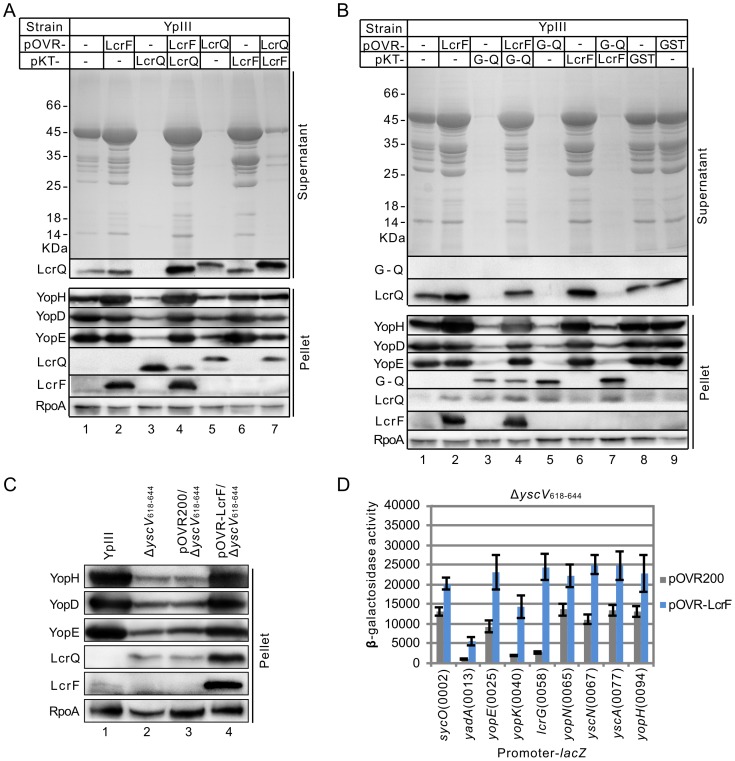
Effects of co-expression of LcrF and LcrQ on T3SS. (A) Secretion and expression of Yops and LcrQ in strains with overexpression of LcrF or LcrQ or co-expression of these two proteins. (B) Effects of co-expression of GST-fused LcrQ (G-Q) with LcrF on Yops and LcrQ expression and secretion. (C) Influences of over-expressing LcrF in an *yscV* mutant (Δ*yscV*_618–644_) on expression of Yops. (D) Promoter activities of LcrF targets in Δ*yscV*_618–644_ strains carrying pOVR-LcrF or pOVR200.

Judging from the data we have obtained above, we hypothesized that intracellular LcrQ blocks the regulatory role of LcrF. If so, the expression levels of these two proteins are important for the regulatory process. To confirm this hypothesis, we over-expressed LcrF using the pOVR200 plasmid in the YPIII strain carrying pKT-LcrQ, in which LcrQ protein was also over-expressed by the pKT100 plasmid. As shown in **Fig. 4A**, over-expression of LcrF released the negative regulatory effect of LcrQ in this strain (compared lane 4 with lane 3). In agreement with this observation, the amount of intracellular LcrQ was obviously decreased in LcrF over-expressed strain (**Fig. 4A**). Therefore, these results can be explained by intracellular amount balance of LcrF and LcrQ proteins. When the amount of LcrF exceeds LcrQ, LcrF dominates the regulatory effect. To prove our hypothesis, we next exchanged the plasmid to over-express these two proteins. pOVR200, which contains a strong promoter, was used to over-express LcrQ and pKT100 was used to express LcrF. In this case, the amount of intracellular LcrQ protein was slightly increased (**Fig. 4A**, compared lane 7 with lane 4), which in turn greatly inhibited the positive effect of LcrF (**Fig. 4A**, compared lane 7 with lane 5). All together, these results suggest that the intracellular amount of LcrQ is important for blocking the regulatory role of LcrF.

To further confirm our hypothesis, we used the pOVR200 plasmid to express GST-LcrQ (which is known to abolish transport of LcrQ [6], [33]) in YPIII strain carrying the pKT-LcrF plasmid. As anticipated, GST-LcrQ is not secreted (**Fig. 4B**). And over-expression of LcrF by the pKT100 plasmid in this background did not show obvious effect on the expression or secretion of Yops (**Fig. 4B**, compared lane 7 with lane 5). In our previous work, we showed that mutation of the *yscV* gene (Δ*yscV*_618–644_), which encodes a component of secretion machinery, accumulates the intracellular LcrQ protein and therefore represses the expression of Yops [9]. We next over-expressed LcrF protein in this background and found that both Yops expression and the promoter activities of its targets were all activated comparing with Δ*yscV*_618–644_ carrying the control plasmid pOVR200 (**Fig. 4C&D**). Taken together, our data suggest that the amount of intracellular LcrQ and LcrF is vital for the regulation of Ysc-Yop T3SS. We hypothesize that intracellular LcrQ abolishes the role of LcrF at 37°C under Ca^2+^ rich conditions, and this inhibition is released when LcrQ is secreted outside of cells under Ca^2+^ depleted conditions.

### LcrQ does not directly interact with LcrF

**Figure 5:**
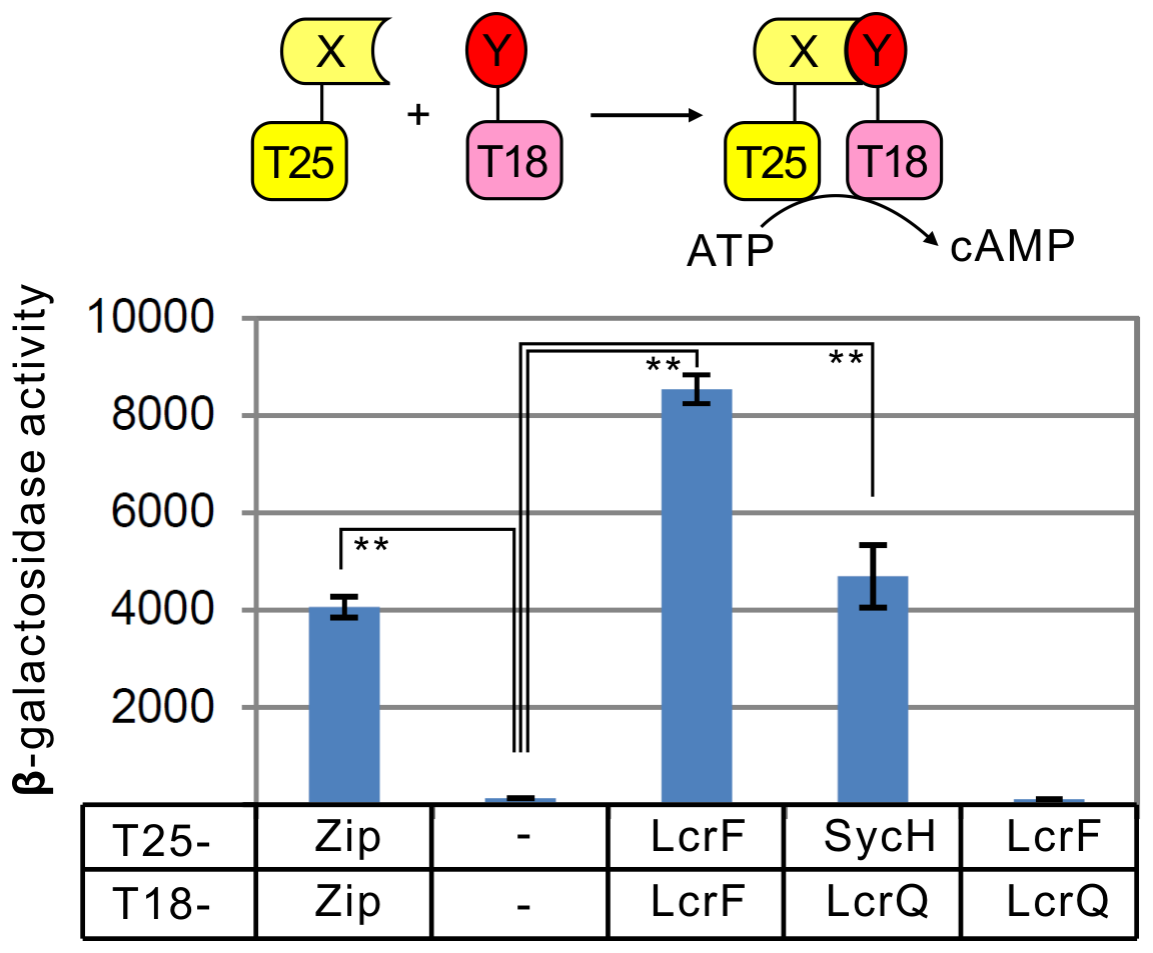
Interaction between LcrQ and LcrF by a bacterial two-hybrid assay. Model for bacterial adenylate cyclase two-hybrid assay is shown in the upper panel. Interaction between LcrQ and LcrF as detected by bacterial two-hybrid system is shown in the lower panel. ** p<0.01.

The regulatory mechanism for the LcrQ protein has not been fully understood. No DNA binding domain can be found on this protein [25], suggesting LcrQ does not directly interact with its target DNA. To explore how LcrQ functions in the regulatory process of LcrF, we applied the Bacterial Adenylate Cyclase Two-Hybrid (BACTH) system to detect possible interaction between these two proteins. As shown in **Fig. 5**, LcrF can form dimer as reported for the AraC family proteins [16], and LcrQ can interact with its chaperone protein SycH [6], [7], which suggest the expression of these two proteins in the BACTH system is stable. However, we could not detect direct interaction between LcrF and LcrQ (**Fig. 5**), suggesting that other factor(s) is also required for LcrQ to interact with LcrF to block its regulatory role.

## Discussion

**Figure 6:**
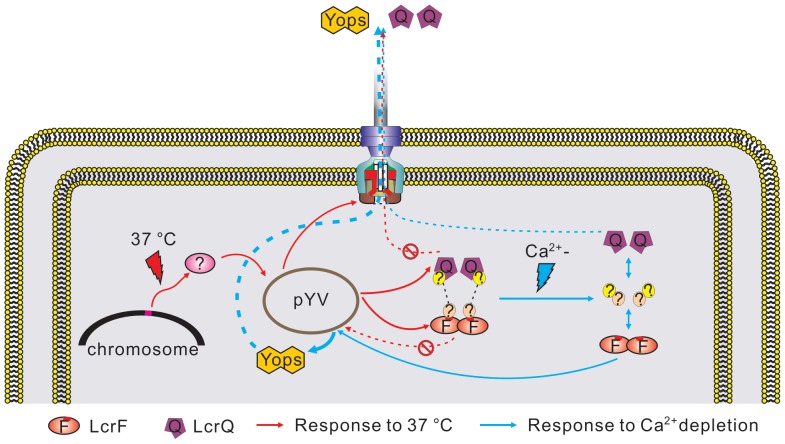
Proposed model for the transcriptional responses of the T3SS genes in *Yersinia*.

The Ysc-Yop T3SS is a strictly regulated system and several modules have been demonstrated to play important roles in this regulatory process [34]. However, this regulation at the transcriptional level has not been systematically analyzed. In this study, we have cloned 97 non-coding fragments from the pYV plasmid into the *lacZ* fusion plasmid and compared the promoter activities of all these fragments under T3SS inducible and non-inducible conditions. The Ysc-Yop T3SS is induced by low Ca^2+^ signal at 37°C *in vitro* [4]. In agreement with this phenotype, transcription of genes on the pYV plasmid is also regulated by low Ca^2+^ signal at 37°C. Based on the promoter activity analyses, we proposed a model for transcriptional regulation of Ysc-Yop T3SS in *Yersinia*. As shown in **Fig. 6** **,** factors encoded on the chromosome first sense the temperature changes. At 37°C, these factors will activate the transcription of most genes on the pYV plasmid, including the secretion machinery components and two regulators LcrF and LcrQ. Under this condition, LcrQ blocks the role of LcrF. Therefore, the expression of T3SS genes could not be massively activated by LcrF at 37°C when LcrQ and LcrF are both intracellularly located. However, the low Ca^2+^ signal will trigger the secretion of LcrQ, which subsequently release the repressive effect of LcrQ, the positive regulatory role of LcrF will thus be exerted.

LcrF and LcrQ are two characterized regulators for Ysc-Yop T3SS in *Yersinia*. LcrF is a classical transcriptional regulator that can directly binds to several promoters on the pYV plasmid [20]. In this study, we systematically characterized the targets of LcrF and identified 9 targets in YPIII. Being consistent with other reports in *Y. enterocolitica* [20], *yopE*, *yopH, yopQ/yopK, yadA, lcrGVHyopBD* operon and *virC* are all activated by LcrF. VirF is not essential for transcription of both *virA* and *virB* operons in *Y. enterocolitica* [15], but these two operons were both regulated by LcrF in *Y. pseudotuberculosis.* Binding of LcrF to these two promoter fragments were confirmed by *in vitro* gel-retardation assay (**Fig. S4**), which suggests these two operons are also the direct targets of LcrF. The regulation of LcrF to its targets is not affected by Ca^2+^, but the transcription of all these genes in YPIII are induced by low Ca^2+^ signal. These data suggest that some other factors are also involved in activating the role of LcrF under low Ca^2+^ conditions. The role of LcrQ in T3SS regulation has not been documented. LcrQ has been proposed to form a complex with YopD-LcrH proteins [35]. A recent study has shown that YscM1 (a homologue of LcrQ in *Y. enterocolitica*) binds to ribosomes together with YopD-LcrH and subsequently inhibits translation of the *yop* genes [36]. Our current study suggests another mechanism for the regulatory role of LcrQ to block the role of LcrF. This blocking process is not in a direct way since we did not observe any direct interaction between these two proteins by a customized bacterial two-hybrid assay, which is in agreement with a previous study showing that LcrQ does not act as an anti-LcrF protein [25]. We suppose that other uncharacterized proteins may act as a linker between these two proteins in this process (**Fig. 6**). Functional homologues of LcrF and LcrQ are found in other pathogens. For example, the T3SS in *Pseudomonas aeruginosa* is triggered by low Ca^2+^ at 37°C and is also globally regulated by two proteins with opposing effects: the AraC family positive regulator ExsA and the secreted negative regulator ExsE [37]. Similar to LcrQ and LcrF, ExsE blocks the role of ExsA by indirect interactions with two intermediate proteins ExsC and ExsD [37]. Further studies are needed to characterize functional homologues of ExsC and ExsD in *Yersinia* and find the binding proteins of LcrF and LcrQ, which will provide information to detail the mechanism of transcriptional regulation to the Ysc-Yop T3SS.

In addition to regulators encoded by the pYV plasmid, several chromosome encoded proteins are also involved in the regulation of the Ysc-Yop T3SS. For example, YmoA, a chromosome-encoded histone-like protein, directly binds to the promoter region of the *lcrF* gene to inhibit its transcription. The ATP-dependent ClpXP and Lon proteases degrade YmoA when the environment temperature achieved at 37°C, which in turn releases the transcription of *lcrF* gene and subsequently activates the T3SS in *Yersinia* [38]. And the LysR family regulatory protein YtxR competes with LcrF in binding with the promoter regions of *yopE* and *yopH* genes in regulating T3SS [39], [40]. All these factors regulate the Ysc-Yop T3SS indirectly through LcrF protein. In our study, we found that transcription of most of the pYV genes are activated by the chromosome-encoded factors at 37°C and these pYV-encoded genes are not all belongs to the targets of LcrF. These data suggest that chromosome-encoded factor is not one of the above proteins. Previous studies have detected that supercoiling of the pYV plasmid changes at 30°C and 37°C and supposed that this DNA topology change may be the temperature-sensing mechanism for T3SS gene expression [41], [42]. Whether the uncharacterized factors are proteins participated in DNA architecture changes need further studies.

As stated in the introduction, the Ysc-Yop T3SS is regulated at both transcriptional and post-transcriptional levels. In this study, our proposed model is only for the transcriptional regulation of the Ysc-Yop T3SS, which could not explain all the phenotypes we have observed in our study. For example, as suggested in our model, deletion of the *lcrQ* gene would facilitate the role of LcrF to activate the Yops at 37°C in the presence of Ca^2+^, as demonstrated by the fact that blockage to the role of LcrF by LcrQ is diminished in the Δ*lcrQ* strain. However, as shown in **Fig. 3C**, the secretion pattern of the Δ*lcrQ* strain under Ca^2+^ rich conditions is different from the normal secretion pattern, which suggests some other factors may also participate in controlling the secretion pathway of Ysc-Yop T3SS. In addition, the promoter activities of *yopE* and *yopH* remains high in Δ*lcrF* background (**Fig. 3E**), but Western blot results showed the expression of these proteins are undetectable in this strain (**Fig. 3B**), which indicate the regulation also occurs at the post-transcriptional level. Moreover, we found that the thermal induction of *yopE*p-*lacZ* is not dependent on the pYV plasmid, which is different from the results using a *yopE-lacZ* fusion (the *yopE* covers a promoter and a ∼700 bp coding region in this fusion) [19]. These suggest some pYV-encoded elements may inhibit the expression of *yopE* at the post-transcriptional level, e.g., YopD protein [21]. The expression of LcrQ protein was increased in LcrF over-expressed strain (**Fig. 4** & **Fig. S5**) but its promoter activity was not activated by LcrF protein. Transcription is only the first step for gene expression. Regulation at other levels should be considered together to fully clarify the complicated regulatory process of Ysc-Yop T3SS.

Among the 97 cloned non-coding fragments, 68 have promoter activities (summarized in **Fig. S6**), and several of these non-coding fragments located inside an operon, e.g., the non-coding fragment upstream of *yopD* gene (pYV0054), which is located in the *lcrGVH-yopBD* operon, were detected with promoter activity, suggesting the transcriptional regulation of pYV-encoded T3SS is complicated. On the other hand, we did not observe promoter activities of some genes located as the first gene in an operon. This may due to the fact that some fragments we chose do not cover the full promoter regions, since open reading frame annotation is only based on bioinformatics analysis from the NCBI, which may introduce mis-annotation. Nevertheless, the promoter bank we have constructed in this study will facilitate further transcriptional regulation analysis in *Yersinia* and results obtained in this study would provide information for further transcriptional regulation analyses related to the Ysc-Yop T3SS.

## Supporting Information

Figure S1 **The mRNA levels of** ***lcrG*** **and** ***lcrQ*** **genes in YPIII at 26°C, 37°C or 37°C with Ca^2+^ depletion.**
(TIF)

Figure S2 **Regulatory role of LcrF to its targets in the ΔpYV strain in the absence (A) or presence (B) of Ca^2+^ at 37°C.**
(TIF)

Figure S3 **Repressive effect of LcrQ over-expression on promoter activities of** ***yopE*** **p,** ***lcrG*** **p and** ***yopH*** **p.**
(TIF)

Figure S4 **The binding of LcrF protein to** ***lcrG*** **p (A), pYV0053p (B),** ***yopN*** **p (C) and** ***sycN*** **p (D). LcrF protein was added at final concentrations of 0, 0.025, 0.05, 0.1, 0.25, 0.5 and 1** μ**M respectively.**
(TIF)

Figure S5 **Relative level of LcrQ protein in LcrF over-expressed strains (quantified from** **Figure 4A** **).**
(TIF)

Figure S6 **Location of promoters on the pYV plasmid. The non-coding fragments with promoter activities detected in this study were labeled with black “P”, and those located upstream of the first gene in an operon but showed no promoter activity in our test were labelled with grey “P”.**
(TIF)

Table S1 **Primers used in this study.**
(XLS)

Table S2 **Genes induced at 37°C on the pYV plasmid.**
(DOC)

Table S3 **Genes induced by Ca^2+^ depletion at 37°C on the pYV plasmid.**
(DOC)
