# Supplementary figures and images for: LcrQ Blocks the Role of LcrF in Regulating the Ysc-Yop Type III Secretion Genes in Yersinia pseudotuberculosis
Source: PLoS One. 2014 Mar 21;9(3):e92243. doi: 10.1371/journal.pone.0092243 (PMC3962397; doi:10.1371/journal.pone.0092243)

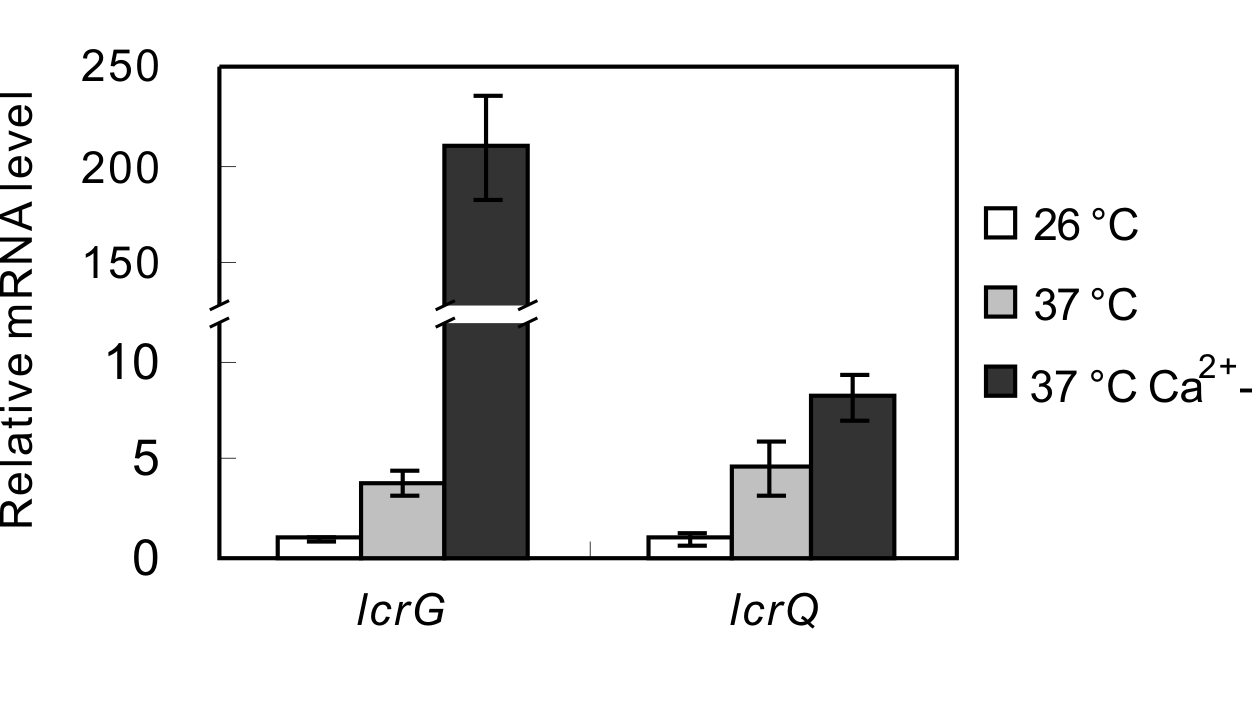

Supplement: Figure S1 — The mRNA levels of lcrG and lcrQ genes in YPIII at 26°C, 37°C or 37°C with Ca2+ depletion. (TIF) [file pone.0092243.s001.tif]

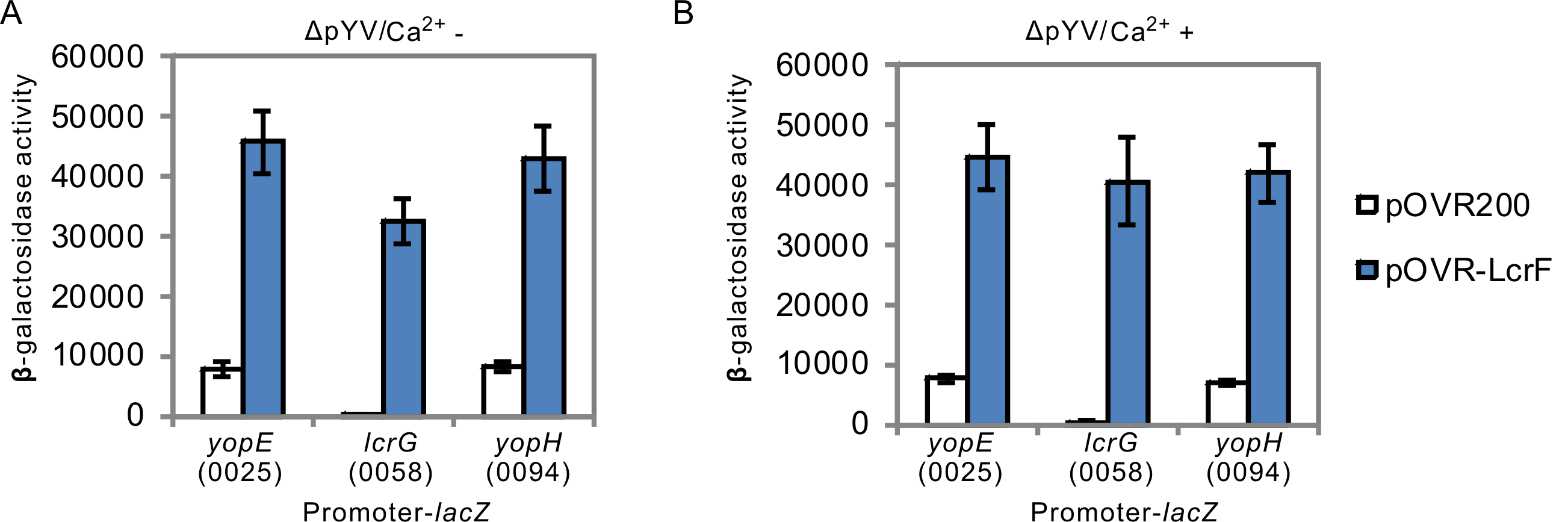

Supplement: Figure S2 — Regulatory role of LcrF to its targets in the ΔpYV strain in the absence (A) or presence (B) of Ca2+ at 37°C. (TIF) [file pone.0092243.s002.tif]

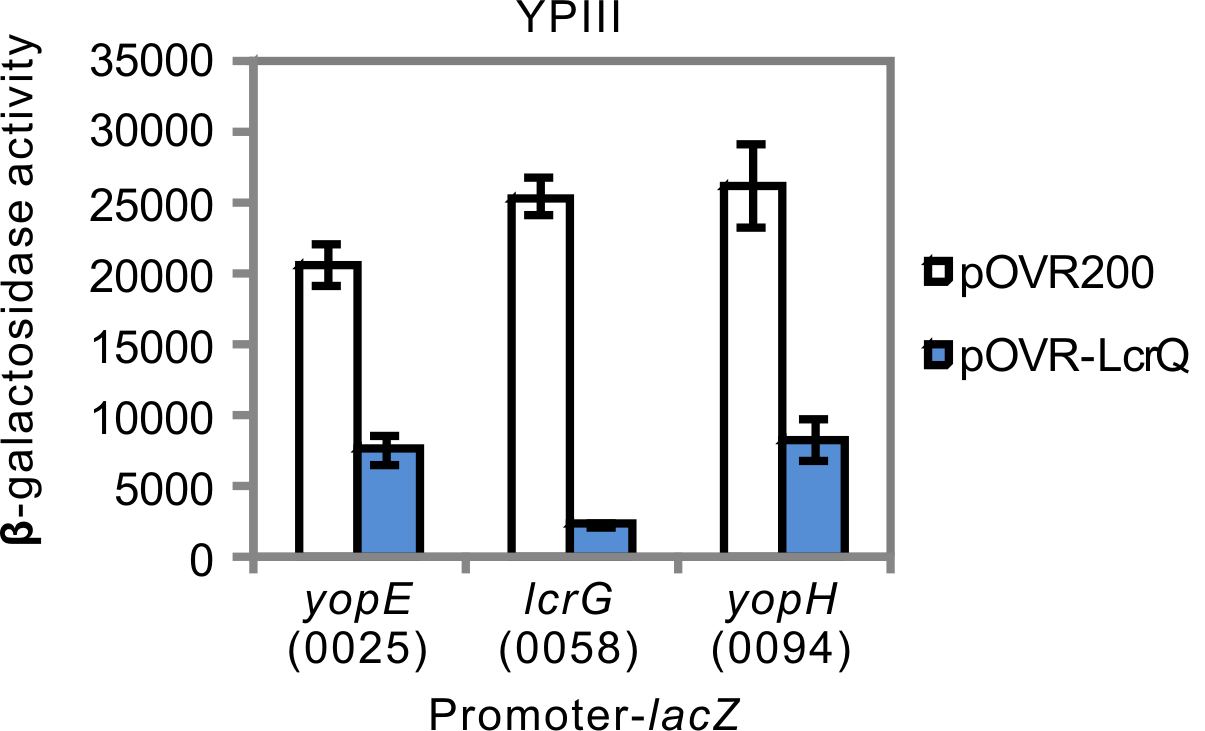

Supplement: Figure S3 — Repressive effect of LcrQ over-expression on promoter activities of yopE p, lcrG p and yopH p. (TIF) [file pone.0092243.s003.tif]

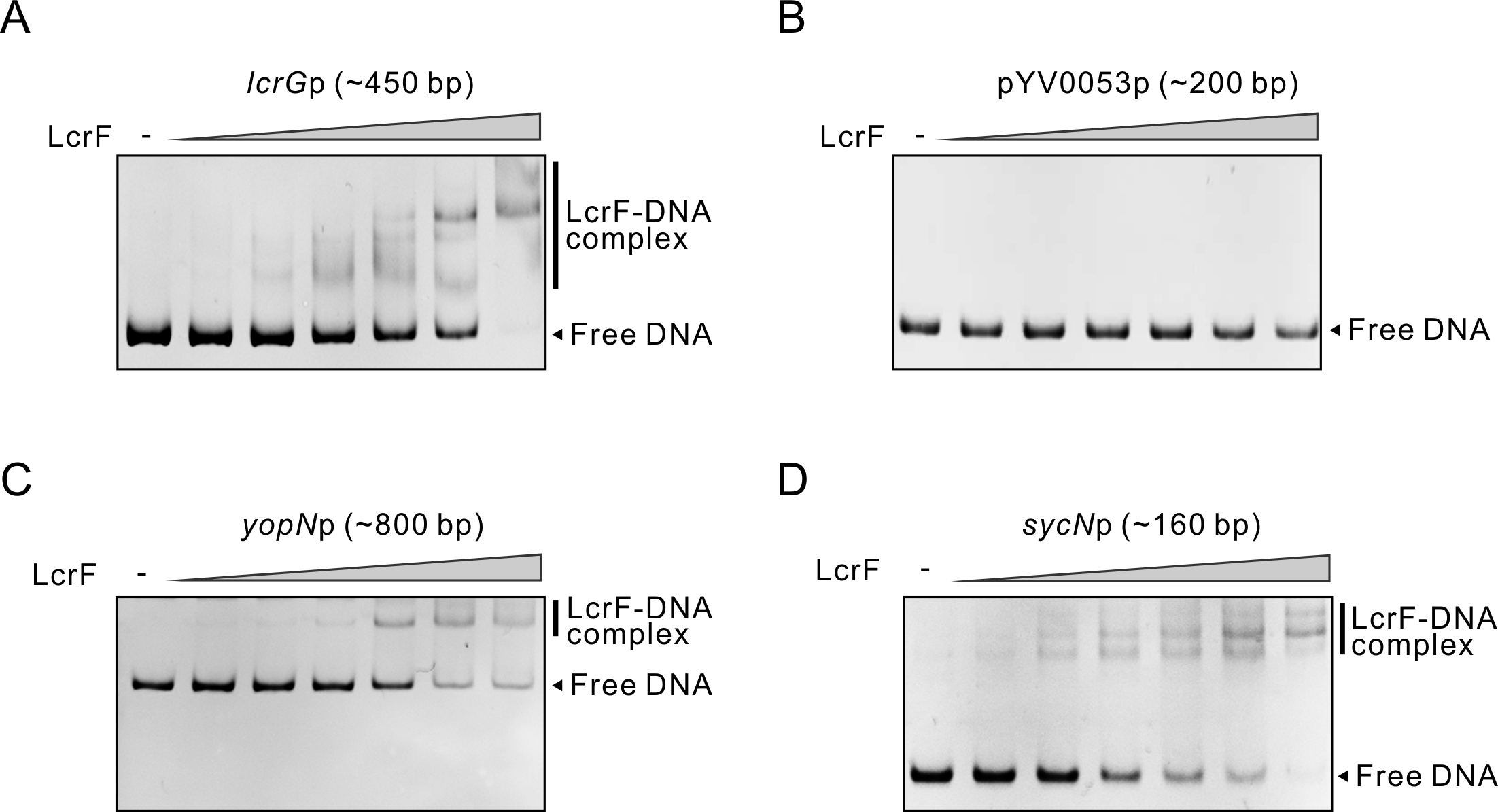

Supplement: Figure S4 — The binding of LcrF protein to lcrG p (A), pYV0053p (B), yopN p (C) and sycN p (D). LcrF protein was added at final concentrations of 0, 0.025, 0.05, 0.1, 0.25, 0.5 and 1 μM respectively. (TIF) [file pone.0092243.s004.tif]

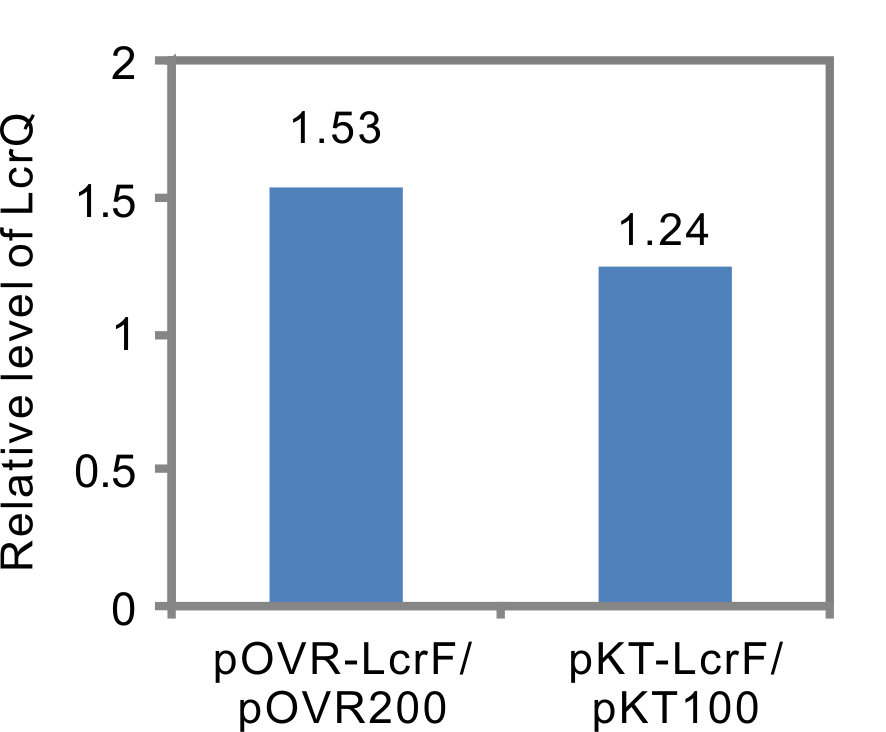

Supplement: Figure S5 — Relative level of LcrQ protein in LcrF over-expressed strains (quantified from Figure 4A ). (TIF) [file pone.0092243.s005.tif]

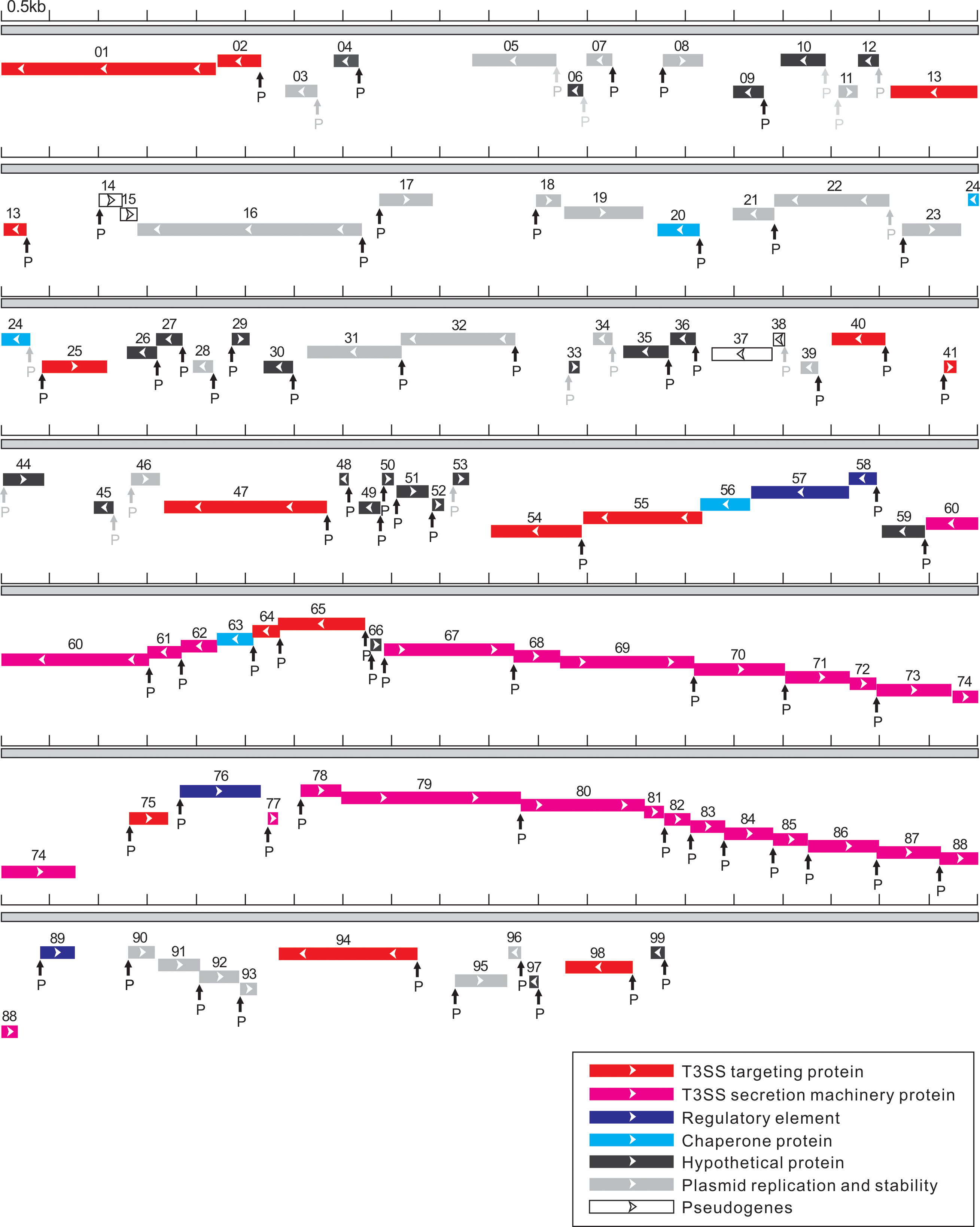

Supplement: Figure S6 — Location of promoters on the pYV plasmid. The non-coding fragments with promoter activities detected in this study were labeled with black “P”, and those located upstream of the first gene in an operon but showed no promoter activity in our test were labelled with grey “P”. (TIF) [file pone.0092243.s006.tif]
