# Supplementary material for: LcrQ Blocks the Role of LcrF in Regulating the Ysc-Yop Type III Secretion Genes in Yersinia pseudotuberculosis
Source: PLoS One. 2014 Mar 21;9(3):e92243. doi: 10.1371/journal.pone.0092243 (PMC3962397; doi:10.1371/journal.pone.0092243)
Supplement: Table S2 — Genes induced at 37°C on the pYV plasmid. (DOC) [file pone.0092243.s008.doc]

**Table S2.** Genes induced by 37 °C on pYV plasmid.

| **pYV number** | **Encoded protein** |
| --- | --- |
| **Plasmid replication** | |
| pYV0016 | tnpA; putative transposase protein |
| pYV0018 | putative transposase |
| pYV0021 | putative transposase |
| pYV0031 | plasmid-partitioning protein |
| pYV0039 | putative transposase |
| pYV0090 | putative transposase |
| pYV0092 | putative transposase |
| **Hypothetical protein** | |
| pYV0002 | hypothetical protein/SycO |
| pYV0004 | hypothetical protein |
| pYV0013 | hypothetical protein/YadA |
| pYV0026 | hypothetical protein |
| pYV0027 | hypothetical protein |
| pYV0029 | hypothetical protein |
| pYV0036 | hypothetical protein |
| pYV0048 | hypothetical protein |
| pYV0050 | hypothetical protein |
| pYV0059 | hypothetical protein |
| pYV0066 | hypothetical protein |
| pYV0099 | hypothetical protein |
| **Secretion machinery components** | |
| pYV0060 | YscV, putative membrane-bound Yop protein |
| pYV0063 | SycN, putative type III secretion protein |
| pYV0067 | Type III secretion system ATPase |
| pYV0068 | YscO, putative type III secretion protein |
| pYV0071 | Type III secretion system protein |
| pYV0073 | YscT, putative type III secretion protein |
| pYV0077 | YscA, putative type III secretion protein |
| pYV0078 | YscB, putative type III secretion protein |
| pYV0080 | YscD, putative type III secretion protein |
| pYV0082 | YscF, putative type III secretion protein |
| pYV0084 | YscH, putative type III secretion protein |
| pYV0086 | YscJ, putative type III secretion lipoprotein |
| pYV0089 | YscM or LcrQ, putative type III secretion regulatory |
| **Effector proteins** | |
| pYV0025 | putative outer membrane virulence protein, YopE |
| pYV0040 | Yop targeting protein YopK |
| pYV0041 | Yop targeted effector YopT |
| pYV0047 | putative targeted effector protein YopM |
| pYV0098 | putative targeted effector protein YopJ; |
