# Supplementary material for: LcrQ Blocks the Role of LcrF in Regulating the Ysc-Yop Type III Secretion Genes in Yersinia pseudotuberculosis
Source: PLoS One. 2014 Mar 21;9(3):e92243. doi: 10.1371/journal.pone.0092243 (PMC3962397; doi:10.1371/journal.pone.0092243)
Supplement: Table S3 — Genes induced by Ca2+ depletion at 37°C on the pYV plasmid. (DOC) [file pone.0092243.s009.doc]

**Table S3.** Genes induced by low Ca^2+^ at 37 °C on pYV plasmid.

| **pYV number** | **Encoded protein** |
| --- | --- |
| **Effector proteins** | |
| pYV0013 | YadA |
| pYV0025 | YopE, putative outer membrane virulence protein |
| pYV0040 | YopK, yop targeting protein |
| pYV0058 | *lcrGVH-yopBD* operon, containing YopB and YopD |
| pYV0065 | YopN, putative membrane-bound Yop targeting protein |
| pYV0094 | YopH; putative protein-tyrosine phosphatase Yop effector |
| **Secretion machinery components** | |
| pYV0067 | Type III secretion system ATPase |
| pYV0077 | hypothetical protein/YscA |
| **Hypothetical protein** | |
| pYV0002 | SycO, hypothetical protein |
